# Complications analysis of Ilizarov bone transport technique in the treatment of tibial bone defects–a retrospective study of 199 cases

**DOI:** 10.1186/s12891-023-06955-0

**Published:** 2023-11-07

**Authors:** Dongwei Feng, Yaxin Zhang, Heping Jia, Guogang Xu, Weize Wu, Fan Yang, Jianan Ding, Dong Li, Kang Wang, Yongjie Luo, Xin Liu, Qi Guo, Zhiguo Zong

**Affiliations:** 1 Department of Pain, The First Affiliated Hospital of Hebei North University, Zhang Jiakou, Hebei China; 2 International Medical Services, The First Affiliated Hospital of Hebei North University, Zhang Jiakou, Hebei China; 3 Department of Otolaryngology and Head Surgery, The First Affiliated Hospital of Hebei North University, Zhang Jiakou, Hebei China; 4 Department of Pathology, The First Affiliated Hospital of Hebei North University, Zhang Jiakou, Hebei China; 5 Department of Joint Surgery, The First Affiliated Hospital of Hebei North University, Zhang Jiakou, Hebei China

**Keywords:** Bone transport, External fixation, Ilizarov technique, Complication, Tibia

## Abstract

**Background:**

The clinical treatment of long bone defets in the extremities caused by trauma, infection, tumours, and nonunion has been a challenge for orthopaedic surgeons. Bone transport techniques have become the only way to treat such bone defects. However, inevitable difficulties and complications related to bone transport techniques have been reported in many studies.

**Aim:**

The purpose of this study was to investigate the risk factors for complications and the effectiveness of the Ilizarov bone transport technique in the treatment of tibial bone defects.

**Methods:**

The study was conducted in 199 patients who underwent treatment with the Ilizarov bone transport technique at our institution from May 2012 to September 2019. Patient demographic data, complications and clinical outcomes after a minimum of 2 years of follow-up were collected and retrospectively analysed. Additionally, a risk factor analysis was performed for the top three major complications. The clinical outcomes were evaluated using the Association for the Study and Application of the Method of Ilizarov (ASAMI) criteria at the last clinical follow-up.

**Results:**

A total of 199 patients underwent follow-up for 12–40 months, with an average of 23.5 months, and all achieved bone healing. A total of 310 complications occurred, with an average of 1.04 minor complications and 0.48 major complications per patient. The top three complications were pin tract infection in 48 cases (61.3%), axial deviation in 86 cases (43.2%), and delayed union in 50 cases (25.13%). Multivariate analysis showed that the bone defect length (*P =* 0.02, *OR* = 5.489), the number of previous surgeries (*P =* 0.003, *OR* = 2.204), and the external fixation index (*P =* 0.01, *OR* = 1.202) were significantly correlated with pin tract infection. Bone defects of the middle 1/3 (*P* < 0.001, *OR* = 23.769), the bone defect length (*P* < 0.001, *OR* = 2.776), and the external fixation index (*P* < 0.001, *OR* = 1.154) were significantly correlated with axial deviation. The bone defect length (*P =* 0.003, *OR* = 1.242), soft tissue defects (*P =* 0.013, *OR* = 0.312) and bone defects of the distal 1/3 (*P =* 0.023, *OR* = 4.257) were significantly correlated with delayed healing. The ASAMI bone score at the last follow-up showed a rate of excellent and good bone results of 95.48% and a rate of excellent functional results of 87.94%.

**Conclusion:**

The Ilizarov bone transfer technique is an effective method for treating tibial bone defects, and shortening the treatment period can reduce the incidence of complications. Older patients and those with longer bone defects, a higher external fixation index, more previous operations, and defects of the middle and distal 1/3 had a higher incidence of complications.

## Background

The clinical treatment of long bone defects in the extremities caused by trauma, infection, tumours, and bone nonunion has been a challenge for orthopaedic surgeons [1]. Although autologous bone transplantation, bone grafting with or without vascular pedicles, allografting and Masquelet techniques have achieved certain clinical results in the treatment of bone defects, they have their own significant limitations [2–4]. With the improvement of external fixation devices and the development of microsurgical techniques, bone transport techniques based on the concept of “distraction osteogenesis” described by Ilizarov have been rapidly promoted at home and abroad because of their simplicity, minimal invasiveness, effectiveness and protective biomechanical environment required for bone healing [5] [6]. However, bone transport treatment is a long process that may cause complications such as pin infection, delayed union and joint stiffness [7] [8], adversely affecting patients’ physiology and psychology and limiting the further promotion and application of this technique.

Previous studies have focused on the prevention and treatment of complications during bone transport, and few studies have analysed overall morbidity and risk factors for complications. We retrospectively analysed 199 cases of tibial bone defects treated with the Ilizarov bone transport technique at our hospital from May 2012 to September 2019 and explored risk factors for complications and the curative effect of the treatment to provide a theoretical basis for better clinical application of bone transport technology.

## Methods

This study was approved by the ethics committee of our institution. Informed written consent was obtained from the participants. In addition, this study was performed in line with the international ethical guidelines for studies involving human subjects according to the Declaration of Helsinki.

### Patients

There are 199 patients with tibial bone defects treated by the Ilizarov bone transport technique from May 2012 to September 2019 in our study. The inclusion criteria were as follows: (1) age between 14 and 65 years; (2) length of the tibial bone defect ≥ 3 cm; and (3) a minimum follow-up of two years after frame removal. The exclusion criteria were as follows: (1) systemic disease, such as liver or kidney insufficiency or disease related to bone metabolism; (2) nerve or blood vessel injury or disease in the affected limb; and (3) poor compliance or loss to follow-up.

During the study period, 205 patients who were treated for tibial bone loss using the Ilizarov bone transport technique were identified. After application of the exclusion criteria, 199 patients were included in the study. There were 171 males and 28 females, with a mean age of 39 years (range, 18–65 years). The aetiology was traumatic bone loss in 40 patients, osteomyelitis in 138 and nonunion in 21.

### Surgical technique

The surgical procedure was planned according to standard anteroposterior (AP) and lateral radiographs of the affected limb. The relevant examination was conducted, surgical contraindications were assessed, and the wound was thoroughly debrided under general anaesthesia or epidural anaesthesia. Prior to bone transport, all hardware was removed, all necrotic and infected bone and soft tissue were subjected to radical debridement, and an antibiotic-impregnated cement spacer was implanted, if necessary, to improve stability. In the case of infection, surface secretions and deep tissue scrapings were retained for bacterial culture and drug sensitivity tests to guide follow-up anti-infection treatment. Cortical bleeding, described by the so-called “paprika sign [9]”, was accepted as an indication of vital osseous tissue. Local tissue flaps or direct tension-free sutures were applied to reconstruct small soft tissue defects, whereas flap transfer or free skin grafting was used to cover larger wounds.

Bone transport was initiated when clinical manifestations and laboratory parameters showed resolution of the infectious process. The type of external fixator was determined by a combination of the location of the bone and soft tissue defects as well as the experience of the surgeon and willingness of the patient. A minimally invasive Gigli saw osteotomy was used to protect the periosteum as much as possible. For bone defects larger than 8 cm or exceeding 40% of the original bone length, a double-level bone transport procedure was performed [10] [11].

### Postoperative management and follow-up

Regular pin-site care. Appropriate antibiotics were administered intravenously for at least 6 weeks until the ESR and CRP level returned to normal based on bacterial culture and drug sensitivity test results. Passive knee and ankle exercises were started on the second postoperative day to encourage early partial weight-bearing. Bone transport was initiated 7–10 days after surgery. For patients treated with flap transfer, bone transport was started after flap healing, which was usually 2–3 weeks. In cases of single-level bone transport, the fragment was transported at a rate of 0.25 mm four times per day. In cases of double-level bone transport, if bone transport was in the same direction (proximal to distal), the fragment near the bone defect was transported at a rate of 0.5 mm four times per day, and the other fragment far from the defect was transported 0.25 mm four times per day. If bone was transported in the opposite direction, each fragment on both sides of the bone defect was transported at a rate of 0.25 mm four times per day. The rate was modified according to the quality of the regenerated tissue on radiography. The frame was removed when the docking site showed union and the lengthening site showed at least three uninterrupted cortices on anteroposterior (AP) and lateral radiographs [12]. Additionally, all patients used a functional brace for 4–6 weeks to protect against refracture.

### Data collection

Demographic and clinical data, including sex, age, number of previous operations, type of external fixation (circular (TrueLok Ring Fixation System, Orthofix, Verona, Italy) or monolateral (Limb Reconstruction System, LRS, Orthofix, Verona, Italy)), size of bone defect, docking time (DT), external fixation time (EFT), external fixation index (EFI) and type of difficulties that occurred during and after the bone transport procedure, were collected. The EFT was defined as the time to removal of the external fixator. The EFI was defined as the ratio of the EFT in days to the size of the bone defect. Radiographic evaluation was conducted every 2 weeks during the bone transport period and monthly in the consolidation phase. All patients underwent close follow-up for a minimum of 2 years after removal of the external fixator.

Complications were classified according to the criteria described by Paley et al [13]. All complications were categorized as minor or major. Minor complications generally required nonoperative treatment or a minor operative procedure that did not have an impact on the fifinal result. Major complication without residual sequelae generally involved a more complex operative procedure that corrected the problem [14]. Bony and functional outcomes were assessed at the last follow-up using the ASAMI [15] score.

### Statistical analysis

Continuous variables (age, size of bone defect, number of previous operations, etc.) were compared by using t tests, and Pearson’s chi-square test or Fisher’s exact test was used to compare categorical variables (sex, type of external fixation, soft tissue defect, location of bone defect and single- or double-level transport). The variables with p < 0.05 in the univariate analysis were entered into the binary logistic regression analysis for analysis of related risk factors, and results with *p* < 0.05 were considered significant. SPSS version 22.0 (IBM Corp, USA) was used to analyse all data.

## Results

A total of 199 patients underwent follow-up for 24–40 months, with an average of 26.5 months. The external fixation time (EFT) was 176 ~ 473 days, with an average of 313 days. The external fixation index (EFI) ranged from 39.76 to 83.5 d/cm, with an average of 53.44 d/cm. All patients achieved bony union. During the course of treatment, there were an average of 1.41 minor complications and 0.48 major complications per patient. The details are shown in Table 1. Among them, 122 patients (61.3%) had a pin tract infection, which was cured in most patients with daily pin site care and oral antibiotics. A total of 3 patients suffered from a deep pin tract infection or pin loosening, which was successfully treated by pin replacement and intravenous antibiotics. Eighty-six patients (43.2%) had axial deviation, and 21 patients with an angle of deviation > 5° underwent correction of the axial deviation by surgery or by the placement of new components. Fifty patients (25.13%) developed delayed union, 37 underwent treatment with the accordion technique, with compression of the docking end to promote bony union, and 13 patients were cured by bone grafting. Forty-two patients (21.11%) developed soft tissue incarceration, which affected callus generation at the docking site in 18 patients; soft tissue resection and segmental trimming were performed, and all patients eventually achieved bony union. Thirty-six patients (18.1%) developed joint stiffness, which was relieved in some patients after removal of the external fixator, and 16 patients recovered after soft tissue release. Eventually, 7 patients had ankle stiffness, and 3 patients had knee stiffness. There were 26 cases of muscle contracture, which improved obviously after passive traction by physical therapy. There were 6 cases of nonunion at the docking site, which were resolved after bone graft internal fixation. There were 5 cases of refracture; 3 of these patients wore a protective brace, 2 underwent bone graft internal fixation, and all achieved bony union. Osteomyelitis recurred in 4 patients and was successfully treated by pin replacement and intravenous antibiotics. The last follow-up evaluation was performed using the ASAMI score, with excellent bone assessment results in 180 cases, good in 10 cases, fair in 4 cases, and poor in 5 cases; the functional assessment results were excellent in 96 cases, good in 79 cases, fair in 20 cases, and poor in 4 cases. The details are shown in Table 1.

**Table 1:** Bone transport-related complications

Complication	Minor	Major
Pin-site infection	119	3
Axial deviation	65	21
Delayed union	37	13
Soft tissue incarceration	24	18
Joint stiffness	20	16
Muscle contractures	16	10
Nonuion	0	6
Refracture	0	5
Osteomyelitis recurrence	0	4
Nerve damage	0	0
Vascular trauma	0	0
Total	281	96

Risk factor analysis of pin tract infection: Univariate analysis showed that single-level transport, the bone defect size, the number of previous operations, the DT, the EFT, and the EFI were associated with pin tract infection. Logistic regression analysis showed that the bone defect length (*P* = 0.02, *OR* = 5.489), number of previous operations (*P* = 0.003, *OR* = 2.204), and EFI (*P* = 0.01, *OR* = 1.202) were significantly associated with pin tract infection. The details are shown in Tables 2 and 3.

**Table 2:** Comparison of pin infection/non- pin infection

Demographic date		pin infection	non-pin infection	*t*/${x}^{2}$	*P*
Sex	Male	102(59.65)	69(40.35)	0.005	0.945
	Female	20(71.43)	8(28.57)		
Type of external fixation	monolateral	103(58.86)	72(41.14)	3.670	0.055
	circular	19(79.17)	5(20.83)		
Soft tissue defect	yes	20(68.97)	9(31.03)	0.254	0.614
	no	102(60.00)	68(40.00)		
Level of bone transport	Single	90(65.94)	47(34.06)	3.567	0.059
	Double	32(50.82)	30(49.18)		
Location of bone defect	proximal 1/3	15(55.56)	12(44.44)	1.048	0.592
	middle 1/3	51(59.30)	35(40.70)		
	distal 1/3	56(65.12)	30(34.88)		
Age		39.70 ± 13.21	38.79 ± 13.67	-0.468	0.640
Previous operation time		3.76 ± 1.34	2.97 ± 1.00	-4.743	P < 0.001
Size of bone defect		7.07 ± 2.67	6.09 ± 1.84	-2.815	0.005
DT		77.89 ± 16.70	69.26 ± 11.78	-4.271	P < 0.001
EFT		338.02 ± 81.19	273.36 ± 49.03	-7.002	P < 0.001
EFI		58.32 ± 11.04	49.93 ± 10.87	-5.254	P < 0.001

**Table 3:** Risk factors of pin-site infection

Variables	β	Standard deviation	Statistical value	*P* value	*OR* value
Size of bone defect	0.508	0.137	13.732	0.000	1.662
Previous operation time	0.558	0.184	9.206	0.002	1.747
DT	0.011	0.017	0.410	0.522	1.011
EFI	0.128	0.027	22.302	0.000	1.137
EFT	0.007	0.004	3.141	0.076	1.007

Analysis of risk factors for axial deviation: Univariate analysis showed that bone defects located in the middle 1/3, the bone defect size, the number of previous operations, the DT, the EFT, and the EFI were associated with axial deviation. Logistic regression analysis showed that bone defects located in the middle 1/3 (*P* < 0.001, *OR* = 23.769), the bone defect length (*P* < 0.001, *OR* = 2.776), and the EFI (*P* < 0.001, *OR* = 1.154) were significantly associated with axial deviation. The details are shown in Tables 4 and 5.

**Table 4:** Comparison of axial deviation /non- axial deviation group

Demographic date		axial deviation	non-axial deviation	t/${x}^{2}$	*P*
Sex	Male	75(42.69)	96(57.31)	0.205	0.651
	Female	11(46.43)	17(53.57)		
type of external fixation	Monoliteral	74(42.29)	101(57.71)	0.512	0.474
	Circular	12(50.00)	12(50.00)		
Soft tissue defect	Yes	14(44.83)	15(55.17)	0.354	0.552
	No	72(42.94)	98(57.06)		
Level of bone transport	Single	61(44.93)	76(55.07)	0.307	0.579
	Double	25(39.34)	37(60.66)		
Location of bone defect	proximal 1/3	9(25.93)	18(74.07)	16.051	P < 0.001
	middle 1/3	51(61.63)	35(38.37)		
	distal 1/3	26(30.23)	60(69.77)		
Age		40.88 ± 13.31	38.19 ± 13.45	-1.414	0.159
Previous operation time		3.73 ± 1.31	3.25 ± 1.21	-2.701	0.008
Size of bone defect		7.56 ± 2.93	6.03 ± 1.70	-4.308	P < 0.001
DT		78.34 ± 17.14	71.67 ± 13.58	-2.967	0.003
EFT		347.16 ± 86.22	287.00 ± 57.40	-5.595	P < 0.001
EFI		57.67 ± 11.52	53.10 ± 11.48	-2.777	0.006

**Table 5:** Risk factors of axial deviation

Variables		β	Standard deviation	Statistical value	*P*	*OR* value
Location of bone defect	distal 1/3			21.686	P < 0.001	
	middle 1/3	2.053	0.451	20.759	P < 0.001	7.790
	proximal 1/3	0.284	0.647	0.193	0.661	1.329
Size of bone defect		0.759	0.148	26.393	P < 0.001	2.135
Previous operation time		0.079	0.158	0.251	0.616	1.083
DT		-0.007	0.017	0.171	0.679	0.993
EFI		0.137	0.030	21.028	0.000	1.147
EFT		0.007	0.004	3.018	0.082	1.007

Risk factor analysis of delayed union: Univariate analysis showed that age, the bone defect length, the DT, the EFT, soft tissue defects, and defects located in the distal 1/3 were associated with delayed union. Logistic regression analysis showed that the bone defect length (*P* = 0.003, *OR* = 1.242), soft tissue defects (*P* = 0.013, *OR* = 0.312), and defects located in the distal 1/3 (*P* = 0.023, *OR* = 4.257) were significantly associated with delayed union. The details are shown in Tables 6 and 7.

**Table 6:** Comparison of delayed union /non- delayed union group

Demographic date		delayed union	non-delayed union	t/${x}^{2}$	*P*
Sex	Male	45(26.32)	126(73.68)	0.915	0.339
	Female	5(17.86)	23(82.14)		
Type of external fixation	monolateral	42(24.00)	133(76.00)	0.977	0.323
	circular	8(33.33)	16(66.67)		
Soft tissue defect	yes	13(10.34)	16(89.66)	7.004	0.008
	no	37(21.76)	133(78.24)		
Level of bone transport	Single	34(21.74)	104(78.26)	2.744	0.098
	Double	16(26.23)	45(73.77)		
Location of bone defect	proximal 1/3	4(14.81)	23(85.19)	6.216	0.045
	middle 1/3	17(19.77)	69(80.23)		
	distal 1/3	29(33.72)	57(66.28)		
Age		43.30 ± 12.33	38.05 ± 13.51	-2.431	0.016
Previous operation time		3.70 ± 1.04	3.38 ± 1.34	-1.562	0.120
Size of bone defect		7.50 ± 2.96	6.42 ± 2.17	-2.374	0.020
DT		78.58 ± 17.52	73.20 ± 14.63	-1.954	0.055
EFT		340.64 ± 81.84	303.75 ± 73.21	-2.991	0.003
EFI		55.50 ± 14.55	52.75 ± 15.54	-1.101	0.272

**Table 7:** Risk factors of delayed union

Variables		β	Standard deviation	Statistical value	*P*	*OR* value
Age		0.025	0.015	3.077	0.079	1.026
Size of bone defect		0.217	0.074	8.685	0.003	1.242
DT		0.008	0.018	0.221	0.638	1.008
EFT		0.007	0.004	3.783	0.052	1.007
Soft tissue defect		-1.166	0.471	6.140	0.013	0.312
Location of bone defect	proximal 1/3			9.126	0.010	
	middle 1/3	0.415	0.651	0.406	0.524	1.514
	distal 1/3	1.449	0.635	5.197	0.023	4.257

## Discussion

In 1989, Ilizarov [16] [17] [18] proposed the bone transport (BT) technique, characterized by the transport of free bone segments to the bone defect area with the aid of an external fixator, followed by eventual mineralization of new bone tissue at the osteotomy site. Compared with traditional techniques, this technique can be used to repair bone and soft tissue defects simultaneously while providing a protected biomechanical environment, which is required for bone healing, and allowing the correction of limb deformities.

The bone transport process includes distraction and docking periods. During the distraction period, the bone segment is generally transported at a rate of 1 mm/d for 7 to 10 days after osteotomy. After reaching the desired extended length, handling is stopped to enter the docking period, which is usually twice as long as the distraction period. The external fixator is removed after complete docking of the new bone. Therefore, large bone defects often require a long EFT, and patient toleration and active cooperation with the treatment are key to treatment success. In this process, anteroposterior and lateral X-rays should be reexamined regularly after the operation to adjust the alignment and monitor the quality of the new bone. In addition, the external fixation device configuration and use of pins are not conducive to early functional exercise in patients, which adversely affects the function of adjacent joints. It has been found that patients with a long EFT may experience psychological disorders such as interpersonal sensitivity, anxiety, and depression, which seriously affect quality of life [19] [20].

In this study, complications such as pin tract infection, axial deviation, delayed union, soft tissue incarceration and joint stiffness occurred. On average, each patient had 1.41 minor complications and 0.48 major complications, and these rates are higher than those reported by Spiegl [21] et al., at 0.88 minor complications and 0.52 major complications on average per patient. This is because osteomyelitis accounts for a relatively large proportion (69%) of complications in our research. However, this issue was finally resolved through various interventions. This article focused on analysing three common complications, as follows.

In this study, pin tract infection (61.3%) was the most common complication, as reported elsewhere, which was addressed by needle tract care, oral antibiotics, and component replacement. The EFI, the number of previous operations, and the bone defect length were independent risk factors for pin tract infection. A larger EFI leads to a prolonged fixation time and increased likelihood of pin tract infection, which is also consistent with previous studies [12]. Yalikun et al. [22] found that an increase in the number of previous operations not only increased bone and soft tissue damage but also may have contributed to recurrent pin tract infection and even bone infection. Furthermore, many operations will not only increase the time spent in bed, inhibit the immune system and increase the risk of osteoporosis but also increase the incidence of pin tract infection. In addition, we found that the length of the bone defect was closely related to the occurrence of pin tract infection, similar to the conclusion reached by Liu [23] et al. in their study. Longer bone defects lead to longer treatment periods and greater tension on soft tissue. Other factors that may affect pin tract infection include the patient’s education level, application of the aseptic concept and the intraoperative technique used for pin fixation [24]. For patients, improving immunity and sanitary conditions can effectively reduce the incidence of pin tract infection. For surgeons, following the principles of low-grade fever and minimally invasive surgery, leaving as much soft tissue as possible at the starting and end points of needle insertion, passing through muscles and ligaments as little as possible, and avoiding osteoporotic sites can also significantly reduce the incidence of pin tract infection.

Antoci [25] found that the anatomical and biomechanical characteristics of valgus and lordosis of the proximal tibia were the main causes of axial deviation in their study. In this study, we found that the bone defect length, the EFT and bone defects located in the middle 1/3 were independent risk factors for axial deviation. The tension on the soft tissue around the bone segment increases with increasing transportation distance, and the gastrocnemius muscle is mainly located posterolateral to the tibia; thus, valgus lordosis often occurs in the bone transport segment [26]. This is caused by tension in the external fixation system at an angle with the force line. In addition, with the increase in the EFT, the connection between the external fixation device and the backbone of the overall structure is prone to micromotion due to the poor biomechanical environment in the segment undergoing bone transport [27]. We also found that bone defects in the middle 1/3 are more prone to axial deviation, which may be caused by the less soft tissue coverage in the middle 1/3 of the tibia. When using a unilateral external fixator, adding a Schanz nail in the bone transport segment and using a reasonable layout of Schanz nails can improve the mechanical stability. In older people, Schanz nails coated with hydroxyapatite can be used to improve the stability of the nail-bone interface. When using circular external fixators, 1200 N is suitable tension for the steel needle; too little tension will reduce the stability of the external fixator, and too much tension may cause the steel nails to break. In this study, the proximal tibial external fixation device was placed as close to the medial and anterior sides as possible.

The incidence of delayed union has been reported to vary widely in the literature but is generally high. There are many reasons for delayed union, and Zhang [28] et al. noted that the blood supply status is related to the quality of osteotylus generation at the docking site. In this study, we also found that bone defects in the distal 1/3 and soft tissue defects were independent risk factors for delayed union. The distal 1/3 contains fewer arteries supplying nutrients, so there is a greater chance of delayed union. Zhong Wan Run [29] applied a bone transport technique to treat 7 patients with bone defects and soft tissue defects, among whom 2 (28.6%) showed nonunion at the docking site. In patients with soft tissue defects, the arterial supply around the bone segments are relatively weak, resulting in poor callus generation at the docking end. In addition, Lavini [30] found that with increasing bone transport distance, fibrosis at the docking site, soft tissue incarceration, and medullary cavity occlusion became more likely, potentially leading to stagnation at the bone segment contact site and finally delayed union or even nonunion. We observed that the length of the bone defect was strongly associated with delayed union. Sala et al. [31] concluded that a poor mechanical environment and small contact area at the segment end of the external fixator also lead to poor bone healing at the docking end. The configuration of the external fixator should be reasonably designed to completely remove the devitalized bone and keep the broken end flat until cortical oozing at the bone end (paprika sign) is observed to avoid the occurrence of delayed union. Although the accordion technique and bone segment compression can stimulate segmental callus regeneration, Teng Xing [32] found that bone segment compression is not effective. Additionally, there are no specific operating standards for the accordion technique. At present, most authors recommend bone grafting at the docking site as soon as possible to promote bone healing [33]. In this study, most patients were treated with bone segment trimming and bone grafting, and all the bones ultimately healed. In addition, the medullary cavity was reconstructed by drilling a Kirschner wire through the segmental end to increase the blood supply to the docking site. However, the operator should strictly evaluate the indications for this method to prevent the occurrence of deep infection.

In the present study, we used the ASAMI scoring system to evaluate the effectiveness of the bone transport method. The rate of excellent and good bone and functional results was 94% and 86.32%, respectively. These results were similar to those of other studies.

A number of factors may contribute to the occurrence of complications during the bone transport procedure. Based on our retrospective study, the defect length, the number of previous operations, the location of the bone defect, soft tissue defects, the docking time, the external fixation time and the external fixation index are statistically significantly associated with the occurrence of complications.

This study is affected by the nature of retrospective studies, the sample size, systemic factors of patients and personalized treatments, as well as other factors. This was a preliminary analysis of the treatment results, without a detailed discussion of the relevant influencing factors or a comparative analysis with other surgical methods. Thus, large, high-quality multicentre randomized controlled studies are needed to further support the findings.

## Conclusion

Our study presents the results of 199 consecutive patients treated using single- or double-level bone transport with particular reference to the complications and their related factors. Bone transport is a reliable method for the reconstruction of bone defects in the tibia with a variety of aetiologies. The key to preventing and reducing complications is to closely monitor the whole process to identify and address problems in a timely and effective manner. At the same time, doctors and patients need to cooperate closely following scientific principles and with regular rehabilitation exercises to reduce the incidence of relevant complications and achieve satisfactory results.

We believe experience has a great impact on the results of different procedures because follow-up and management of expected complications are cornerstones of treatment strategies. Future research should focus on reducing the difficulties associated with long bone transport, such as methods for enhancing the regeneration of bone and reducing pin tract infections. Advances through research to stimulate regeneration and reduce the duration of treatment will revolutionize limb lengthening surgery.

## Data Availability

The datasets analyzed during the current study are available from the corresponding author on reasonable request.
